# Dynamic Changes in Oxidative Stress and Epigenetic Modifications in the Ventral Mesencephalon and Striatum of MPTP-Treated Mice: Implications for Parkinson's Disease Pathogenesis

**DOI:** 10.1007/s12640-025-00748-0

**Published:** 2025-07-04

**Authors:** Pablo Gallo-Soljancic, Maria Egle De Stefano, Ana-Maria Gonzalez-Cuello, Emiliano Fernandez-Villalba, Lode Godderis, Maria Trinidad Herrero

**Affiliations:** 1 Clinical & Experimental Neuroscience (NICE), Department of Human Anatomy and Psychobiology, Institute for Aging Research. School of Medicine, University of Murcia, Campus Mare Nostrum, Murcia, Spain; 2 Institute for Bio-Health Research of Murcia (IMIB-Pascual Parrilla), Campus of Health Sciences, El Palmar-Murcia, Spain; 3 Department of Biology and Biotechnologies “Charles Darwin”, Sapienza University of Rome, Rome, Italy; 4 Center for Research in Neurobiology “Daniel Bovet”, Sapienza University of Rome, Rome, Italy; 5 Centre for Environment and Health, KU Leuven, Louvain, Belgium; 6 External Service for Prevention and Protection at Work, IDEWE, Louvain, Belgium

**Keywords:** DNA methylation, Epigenetics, MPTP, Parkinson’s disease, 8-OHdG, 5-hmC

## Abstract

This study investigates the effects of an acute 1-metil 4-fenil 1,2,3,6-tetraidro-piridina (MPTP) treatment, a known inducer of parkinsonism, on oxidative stress and epigenetic changes in the mouse ventral midbrain (VM) and striatum. Key markers were analyzed at 4, 8, 24, and 48 h post-injections: the hydroxylated form of the purine guanine (8-hydroxy-2'-deoxyguanosine; 8-OHdG), a marker of oxidative stress; the methylated form of cytosine (5-methylcytosine; 5-mC), associated with gene silencing; the hydroxy methylated form of cytosine (5-hydroxymethylcytosine; 5-hmC), involved in demethylation and gene regulation. The results showed a pronounced decrease in 8-OHdG levels in the VM, suggesting a rapid oxidative stress response, whereas the striatum exhibited a less pronounced response, reflecting regional differences in oxidative stress vulnerability DNA methylation patterns revealed complex and biphasic changes in 5-mC levels in the VM, contrasted with a less pronounced response in the striatum, suggesting disrupted methylation homeostasis and regional epigenetic variability. MPTP treatment also significantly reduced in 5-hmC levels in the VM, pointing to impaired active DNA demethylation and compromised epigenetic flexibility. In contrast, the striatum maintained consistently high 5-hmC levels, reflecting compensatory hydroxymethylation mechanisms specific to this region. These findings highlight pronounced regional differences in oxidative stress vulnerability and epigenetic regulation, with the VM showing heightened sensitivity to oxidative damage and impaired epigenetic flexibility. This underscores the importance of understanding the role of oxidative and epigenetic mechanisms in Parkinson’s disease pathophysiology, The changes pave the way for novel therapeutic strategies targeting oxidative DNA damage and epigenetic homeostasis.

## Introduction

With the increasing the aging population, there has been a parallel rise in the prevalence of neurodegenerative diseases. Among these, Parkinson's disease (PD) stands out as a chronic and progressive disorder, ranking as the second most common neurodegenerative disease after Alzheimer's (Pringsheim et al. 2014; Abdullah et al. 2015; Cuenca et al. 2019). PD primarily affects dopaminergic neurons in the substantia nigra, leading to a range of motor and non-motor symptoms that significantly impact patients'quality of life (Cuenca et al. 2019). Its prevalence increases with age, affecting about 1–2% of individuals over 60 and rising to 3–5% among those aged 85 to 89. By 2040, the number of PD cases is projected to double from the 6.2 million reported in 2015, underscoring its significance as a global public health challenge with substantial economic implications (Pringsheim et al. 2014; Chaudhuri et al. 2024).

A hallmark of PD is the presence of Lewy bodies, intracellular protein aggregates primarily composed of misfolded α-synuclein (Cuenca et al. 2019). Disease progression typically follow a specific pattern, beginning in the olfactory bulbs and dorsal motor nucleus of the vagus nerve, then advancing to the locus coeruleus and substantia nigra, and eventually affecting cortical brain regions (Wakabayashi 2020). This progression accounts for the early onset of non-motor symptoms, such as olfactory deficits and gastrointestinal dysfunction, which often precede motor symptoms by years (Bourdenx et al 2020; Torres-Pasillas et al. 2023; Tan et al. 2023). The characteristic motor symptoms include resting tremor, rigidity, bradykinesia, and postural instability while non-motor symptoms such as sleep disturbances, depression, anxiety, cognitive impairment, and autonomic dysfunction significantly contribute to the disease burden (Goldman and Postuma 2014).

Despite advances in understanding PD, significant gaps remain in our knowledge of how epigenetic changes and oxidative stress contribute to neurodegeneration (Hirsch and Hunot 2009; Kaut et al. 2022; Tsalenchuk et al. 2023). Current research has increasingly focused on epigenetic modifications, such as DNA methylation and histone changes, in relation to PD pathogenesis. Studies have demonstrated connections between PD and epigenetic alterations, (Kochmanski et al. 2019; Tsalenchuk et al. 2023), particularly in the methylation of 5-methylcytosine (5-mC) and the hydroxymethylation of 5-hydroxymethylcytosine (5-hmC) in cortex, whole blood and saliva samples from PD patients (Chuang et al. 2017; Henderson et al. 2021; Kaut et al. 2022). These changes may serve as biomarkers for early diagnosis and therapeutic targets for treatment development.

Animal models have been pivotal in studying PD mechanisms and developing therapeutic strategies (Dauer and Przedborski 2003; Dovonou et al. 2023; Lal et al. 2024). Among these, the 1-Methyl-4-phenyl-1,2,3,6-tetrahydropyridine hydrochloride (MPTP)-induced mouse model is widely used, as it replicates key pathological and clinical features of PD in mice (Przedborski et al. 2001) and in monkeys (Kastner et al. 1994; Perez-Otano et al. 1992). MPTP is a neurotoxin that selectively induces dopaminergic neurons death in the substantia nigra, mimicking human PD pathology (Przedborski et al. 2001; Dauer and Przedborski 2003; Lal et al. 2024). This model has facilitated research on neurodegeneration and the evaluation of potential therapeutic (Du et al. 2001; Gil-Martinez et al. 2020a; Costa et al 2021; De Araújo et al. 2022; Du and Bu 2021; He et al. 2022).

Preclinical studies have confirmed that oxidative stress is a central factor in PD pathogenesis (Faucheux et al. 1995; Richardson et al. 2005; Perier et al. 2003; Callio et al. 2005; Gmitterová et al. 2009; Bolner et al. 2011; Vastegani et al. 2023). The accumulation of reactive oxygen species (ROS) damages lipids, proteins, and DNA (Jiménez-Jiménez et al. 2016; Trist et al. 2019; Aborode et al. 2022). Elevated levels of 8-hydroxy-2'-deoxyguanosine (8-OHdG), a marker of oxidative DNA damage, have been detected in the urine, plasma, and cerebrospinal fluid of PD patients (Gmitterová et al. 2009; Bolner et al. 2011). Measuring 8-OHdG provides insights into oxidative damage and the effectiveness of antioxidant therapies (Bogdanov et al. 2008).

However, despite advances in understanding the etiopathogenesis and pathophysiology of PD, significant gaps remain regarding the timing and mechanisms by which epigenetic changes and oxidative stress contribute to neurodegeneration in relation to PD onset and progression. This study aims to address these gaps by investigating the levels of 8-OHdG, 5-mC, and 5-hmC in the ventral mesencephalon (VM) and striatum of MPTP-treated mice. By examining the effect of MPTP on these markers, the study seeks to elucidate the relationship between oxidative stress, epigenetic modifications, and dopaminergic neuron degeneration. Although our study has been conducted on brain samples, the findings aim to confirm the potential of these biomarkers for early, non-invasive PD diagnosis through saliva or blood samples, and to explore their utility as therapeutic targets.

## Materials and Methods

### Animal Model and Ethical Statement

This study was conducted on 40 three-month-old male C57BL/6 J mice (25–30 g body weight; b.w.) purchased from Charles River Laboratories (Janvier Labs, Le Genest Saint Isle, France). The mice were housed under temperature-controlled conditions (21 ± 1 °C) with a 12-h light/dark cycle. The study strictly adhered to the"Three R's principle”, and all animal care, housing and experimental procedures complied the European Community Council Directive (2010/63/UE) for the use of animals in preclinical research. The protocols were approved by the Institutional Committee on Animal Ethics at the University of Murcia (REGA ES300305440012).

### Experimental Design and MPTP Administration

**Fig. 1:**
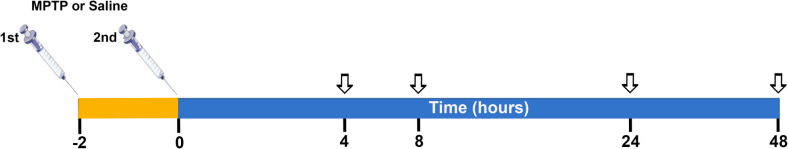
Experimental design for MPTP-induced parkinsonism in mice. Parkinsonism was induced by two intraperitoneal injections of 1-methyl-4-phenyl-1,2,3,6-tetrahydropyridine hydrochloride (MPTP) (30 mg/kg b.w.), administered 2 h apart. Control mice received an equivalent volume of saline (150–180 µl, depending on b.w.) and were injected in parallel. In the diagram, block arrows indicate the four time points (4, 8, 24, and 48 h) at which saline- and MPTP-injected mice were sacrificed after the second injection

Mice were randomly divided into two groups: the control group (n = 20) and the MPTP-treated group (n = 20). Parkinsonism was induced via two intraperitoneal (i.p.) injections of MPTP HCl (30 mg/kg, freshly diluted in saline) (Merck Life Science S.L.U., Madrid, Spain; Cat. No. M0896), administered 2 h (hrs) apart in a volume of 150–180 µl, depending on b.w., following established protocols (Gil-Martinez et al. 2020a; Barcia et al. 2011, 2012). Control animals received vehicle (saline) injections according to the same schedule. Following MPTP intoxication, mice were housed in a quiet, temperature-controlled room and provided with cotton wool to minimize discomfort, in accordance with the safety protocol for MPTP treatment. Mice were sacrificed at 4, 8, 24, and 48 h post-MPTP or saline administration (Fig. 1) via cervical dislocation under an overdose of ketamine (50 mg/Kg, Imagene, Merial) and Xylazine (50 mg/Kg, Xilagesic, Calier Laboratories). These time points were selected to evaluate the acute (4 and 8 h) and early subacute (24 and 48 h) effects of the treatments, providing insights into the progression of MPTP-induced neurotoxicity in this Parkinson’s disease model. Within each group, 5 mice were assigned to each of the four time points. Random assignment ensured that there was no bias in the distribution of animals across the different sacrifice times. Brains were immediately removed, and the ventral mesencephalon (VM) and striatum were dissected and stored at −80°C until use.

### Sample Preparation

Approximately 40–90 mg of each sample was homogenized in lysis buffer (100 mM Tris–HCl, 200 mM NaCl, 1 mM EDTA, 2 mM DTT, 0.05% triton, one tablet of Complete Protease Inhibitor Mix (Roche, Cat. No. 11836170001) and one tablet of PhosSTOP Phosphatase Inhibitor Cocktail (Roche, Cat. No. 4906837001) at a ratio of 1 g tissue per 9 ml buffer, to maintain sample integrity during processing. Homogenization was performed using a mini-Bead-Beater (BioSpec Products, Bartlesville, OK, USA), with three cycles of 30 s (sec) each, separated by with 5-min (min) cooling intervals on ice. After the final homogenization, samples were incubated on ice for 30 min and then stored at −80◦C until use.

### DNA Extraction

For DNA extraction, an aliquot of the homogenized sample was mixed with RLT Plus buffer from the AllPrep DNA/RNA Mini Kit's (QIAGEN®, Hilden, Germany) in a 1:3 ratio to ensure proper lysis and DNA preservation. The lysate was processed through an AllPrep DNA Mini spin column, allowing selective DNA binding to the column membrane. Multiple wash steps were performed using kit-provided buffers to remove contaminants such as residual proteins and inhibitors. Purified DNA was eluted in a low-salt buffer and its concentration and purity were assessed spectrophotometrically. This adapted protocol enabled the extraction of high-quality genomic DNA from samples previously prepared for protein analysis, ensuring compatibility with subsequent epigenetic analyses. DNA concentration and purity were assessed spectrophotometrically, with an average 260/280 ratio of approximately 1.8. All samples were normalized to the same DNA concentration prior to performing the assays to ensure the comparability of results. Positive and negative controls provided in the assay kits were included in every run to validate the assay performance and correct for any interplate variations.

### Quantification of 8-Hydroxy-2'-Deoxyguanosine

8-Hydroxy-2'-deoxyguanosine (8-OHdG) levels were quantified using the EpiQuik 8-OHdG DNA Damage Quantification Direct Kit (Colorimetric) (Epigentek Group Inc®, Farmingdale, USA; Cat. No. P-6008). Approximately 200 ng of DNA samples (1–8 µl, depending on the initial DNA concentration) and 100 ng of standard containing known amounts of 8-OHdG, were loaded onto a 96-well plate according to the kit recommendations and incubated with a primary antibody specific for 8-OHdG. After washing, a secondary antibody was applied, followed by additional washes and colorimetric substrate development. The absorbance was measured using a Multiskan GO microplate reader (Thermo Fischer Scientific, Waltham, MA USA), and results were quantified against a standard curve of known 8-OHdG concentrations. Data were expressed as the percentage of 8-OHdG relative to the total guanine content in the DNA samples.

### Quantification of 5-Methylcytosine

Total 5-Methylcytosine** (**5-mC) content in genomic DNA was measured using the MethylFlash Global DNA Methylation (5-mC) ELISA Easy Kit (Colorimetric) (Epigentek Group Inc®, Farmingdale, USA; Cat. No. P-1030). Briefly, 90 ng of DNA samples (2–4 µl, depending on the initial DNA concentration) and 100 ng of standard containing known amounts of 5-mC, were loaded in triplicate onto a 96-well streptavidin-coated plate and incubated at room temperature (RT) for 60 min with mild agitation (~ 100 rpm). Unbound DNA was removed with multiple washes using 1X Wash Buffer (200 µl per wash). A blocking buffer (200 µl per well) was added, followed by a 30 min incubation at RT, and successively wells were incubated with a primary antibody against the 5-mC (1:100 dilution, 100 µl per well) for 60 min at RT, washed three times with buffer, and then incubated with a horseradish peroxidase-conjugated secondary antibody. After a thorough wash with buffer, a Developing Solution (100 µl per well) was added, followed by a stop solution, and absorbance of the colorimetric reaction was measured at 450 nm (reference wavelength of 655 nm) using a Multiskan GO microplate reader. Results were expressed as the percentage of 5-mC relative to the total cytosine content.

### Quantification of 5-Hydroxymethylcytosine

Total 5-Hydroxymethylcytosine (5-hmC) content of genomic DNA was determined using the MethylFlash Hydroxymethylated DNA Quantification Kit (Colorimetric) (Epigentek Group Inc®, Farmingdale, USA Cat. No. P-1032). Briefly, 100 ng of DNA samples (2–4 µl, depending on the initial DNA concentration) and 100 ng of standard containing known amounts of 5-hmC, were loaded in duplicate onto a multi-well plate pre-filled with 80 µl of binding solution per well, and incubated at 37 °C for 90 min with mild agitation (~ 100 rpm). Unbound DNA was removed via three washes with 1X Wash Buffer (150 µl per well/wash). A primary anti-5-hmC antibody (1:1,000 dilution; 50 µl per well) was added and incubated at RT for 60 min. After three washes with buffer, a secondary antibody (1:2,000 dilution; 50 µl per well) was added and incubated for 30 min at RT. Following four washes, an enhancer solution (50 µl per well) was added and incubated for 30 min. The plate was washed five times with 1X Wash Buffer and once with 1X phosphate-buffered saline (150 µl per well/wash), a fluorescent development solution was added (50 µl per well) was added, and the relative fluorescence intensity (RFU) was measured using a Multiskan GO microplate reader. Results were expressed as the percentage of 5-hmC relative to total DNA.

### Statistical Analysis

All data were expressed as the mean ± standard deviation (SD). To assess the effect of MPTP and saline treatments on 8-OHdG, 5-hmC, and 5-mC levels in the VM and striatum, a two-way ANOVA followed by Tukey’s multiple comparison test was performed. Data normality was verified prior statistical analysis. All analyses were conducetd using GraphPad Prism 8.0 (GraphPad Software, San Diego, California). A p-value of ≤ 0.05 was considered statistically significant.

## Results

### Dynamics of 8-Hydroxy-2'-Deoxyguanosine in the Ventral Mesencephalon and Striatum Following MPTP Treatment

**Fig. 2:**
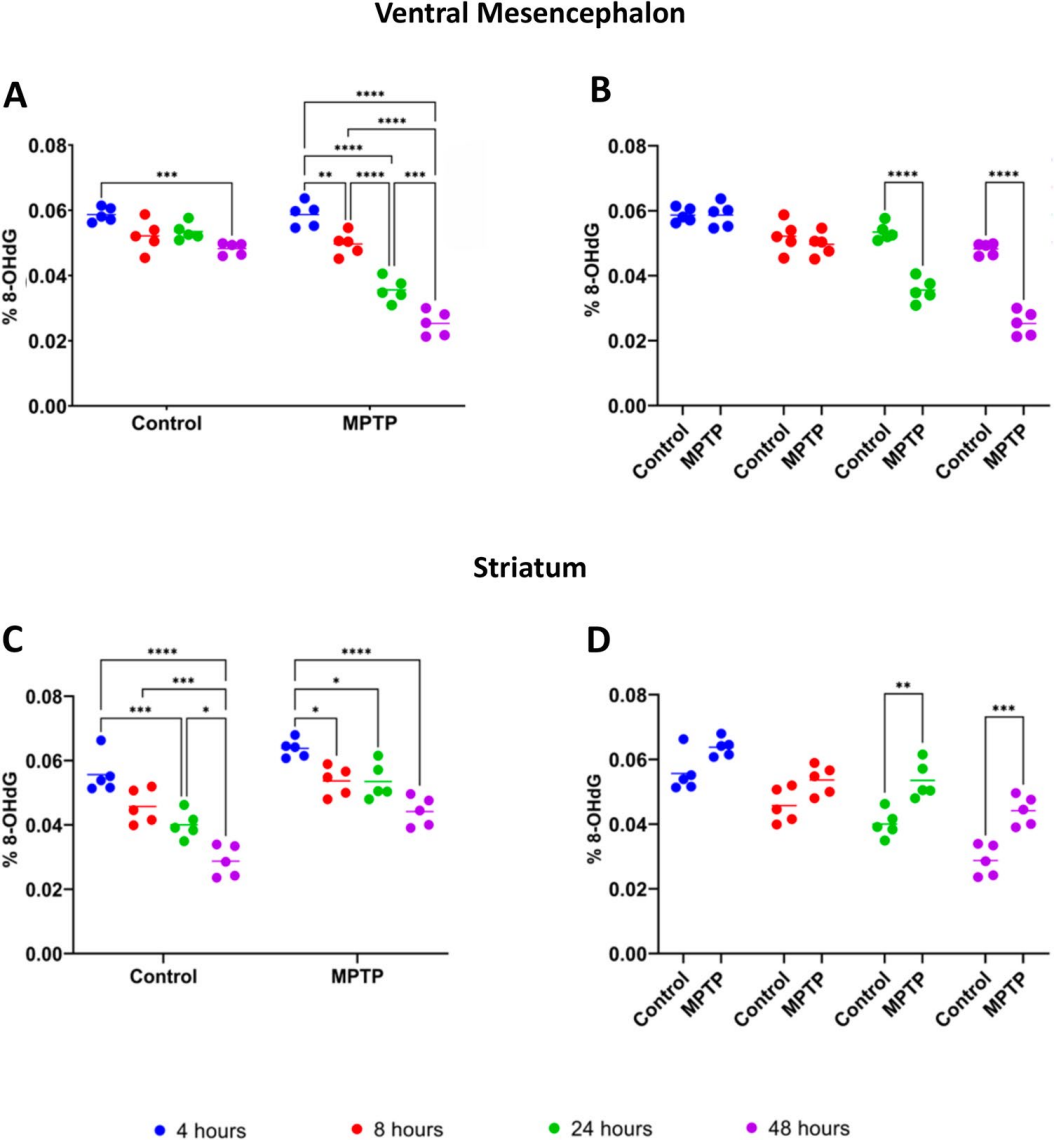
Levels of 8-hydroxy-2'-deoxyguanosine (8-OHdG) in the ventral mesencephalon (**A**,**B**) and striatum (**C**,**D**) of saline (control) and MPTP-treated mice. **A** Evaluation of 8-OHdG levels in the ventral mesencephalon within each experimental group across the different time points after. **B** Comparison of the 8-OHdG levels in the ventral mesencephalon between control and MPTP-treated groups at the different time points. **C** Evaluation in 8-OHdG levels in the striatum within each experimental group across the different time points after. **D** Comparison of the 8-OHdG levels in the striatum between control and MPTP-treated groups at the different time points. Data are expressed as the mean ± SD expressed as the percentage of 8-OHdG relative to the total guanine content in the DNA samples, and evaluated for statistical significance by a two-way ANOVA followed by Tukey’s multiple comparison test. *p ≤ 0.05, **p < 0.01, ***p < 0.001, ****p < 0.0001

In saline-injected mice (control group), a significant decrease in 8-OHdG levels was observed only between the first time point (4 h) and the last (48 h) post-treatment (Fig. 2A) (*p* < 0.001). This suggests a reduction in oxidative DNA damage over time under normal physiological conditions, potentially due to repeated injections. In contrast, the MPTP-treated group exhibited a more diversified and pronounced pattern of changes in 8-OHdG levels. A significant decrease was observed between 4 and 8 h post-treatment (*p* < 0.01), indicating an early and rapid response to MPTP (Fig. 2A). The reduction became even more pronounced between 4 and 24 h, and 4 and 48 h (Fig. 2A) (*p* < 0.0001), suggesting a sustained and progressive response to oxidative damage. Significant reductions were also noted between 8 and 24 h (*p* < 0.0001), 8 and 48 h (*p* < 0.0001), and 24 and 48 h (*p* < 0.001) (Fig. 2A), indicating the persistence of MPTP effects at later time points. When comparing control and MPTP-treated groups at the same time points, significant differences were observed at 24 h (*p* < 0.0001) and 48 h (*p* < 0.0001) post-treatment, with lower 8-OHdG levels in the MPTP-injected mice (*p* < 0.0001).

In the striatum in control mice, 8-OHdG levels progressively and significantly decreased between 4 and 24 h (*p* < 0.001), 4 and 48 h (*p* < 0.0001), and 8 and 48 h (*p* < 0.001) post-treatment (Fig. 2C). A smaller but still significant reduction was also observed between 24 and 48 h (*p* < 0.05) (Fig. 2C). The temporal profil of 8-OHdG levels in MPTP-treated mice differed not only from the control group, but also from the VM response to the same treatment. In the striatum of MPTP-treated mice, significant decrease in 8-OHdG levels were detected between 4 and 8 h (*p* < 0.05), 4 and 24 h (*p* < 0.05), and 4 and 48 h (*p* < 0.0001) post-treatment (Fig. 2C). However, the reductions in the MPTP group were less pronounced compared to the control group, particularly at earlier time points. As a result, significant differences between MPTP and control mouse groups emerged at 24 h (*p* < 0.01) and 48 h (*p* < 0.001) post-treatment, with lower 8-OHdG levels observed in the MPTP -injected mice compared to controls (Fig. 2D).

### Dynamics of 5-Methylcytosine in Ventral Mesencephalon and Striatum Following MPTP Treatment

**Fig. 3:**
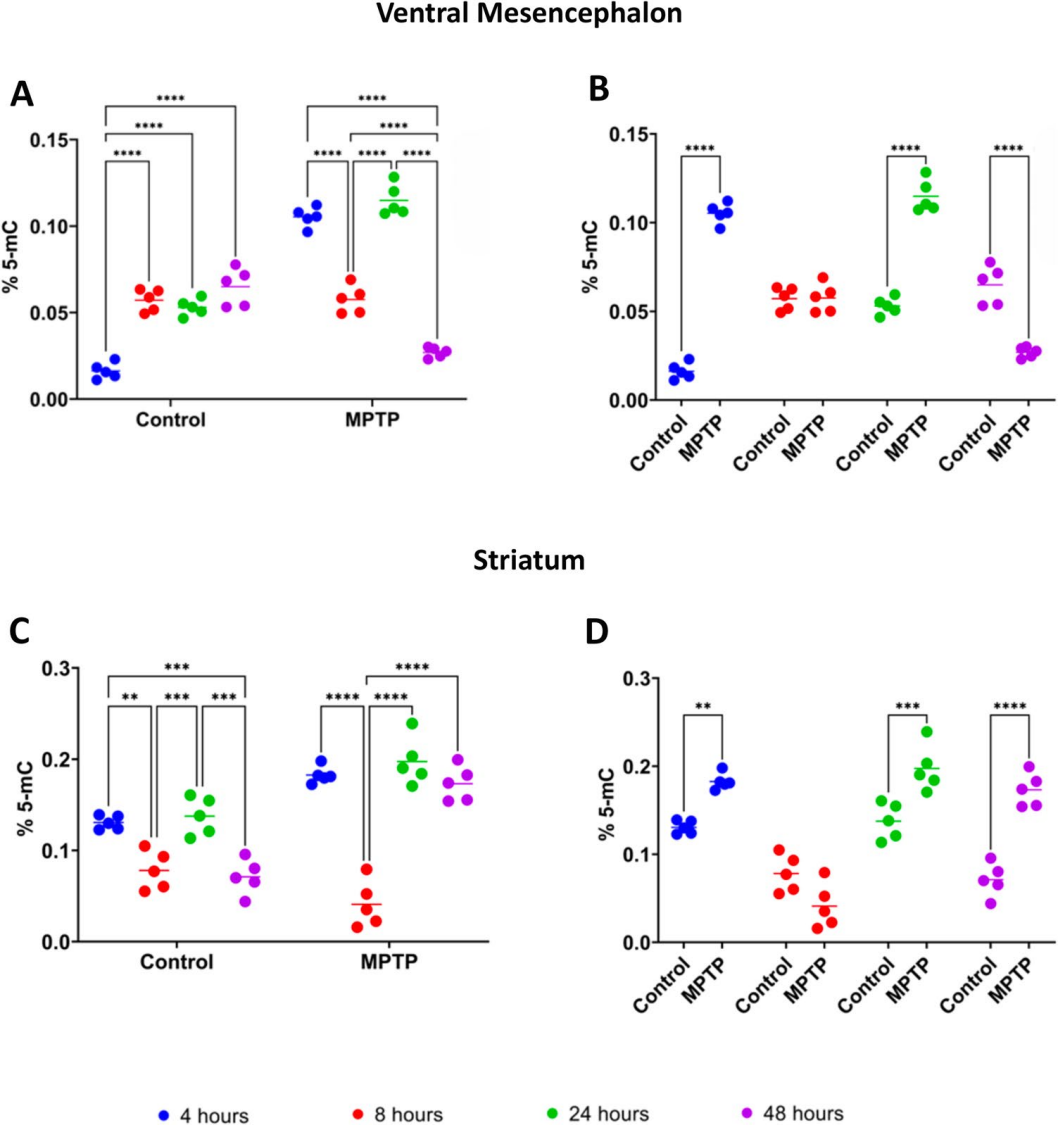
Levels of 5-methylcytosine (5-mC) in the ventral mesencephalon (**A**,**B**) and striatum (**C**,**D**) of saline-(control) and MPTP-treated mice. **A** Evaluation of 5-mC levels in the ventral mesencephalon within each experimental group across the different time points after. **B** Comparison of 5-mC levels in the ventral mesencephalon between control and MPTP-treated groups at the different time points. **C** Evaluation in 5-mC levels in the striatum within each experimental group across the different time points after. **D** Comparison of the 5-mC levels in the striatum between control and MPTP-treated groups at the different time points. Data are expressed as the mean ± SD of the percentage of 5-mC relative to the total cytosine content and evaluated for statistical significance by a two-way ANOVA followed by Tukey’s multiple comparison test. *p ≤ 0.05, **p < 0.01, ***p < 0.001, ****p < 0.0001

In the VM, the control group exhibited a significant early increase in 5-mC levels between 4 and 8 h (*p* < 0.0001), which persisted between 4 and 24 h (*p* < 0.0001), and 4 and 48 h (*p* < 0.0001) post-treatment (Fig. 3A). No significant differences were observed among 8-, 24- and 48-h time points, indicating a rapid increase in DNA methylation under normal physiological conditions, which was maintained over time. In contrast, the MPTP-treated group showed a distinct and complex pattern. 5-mC levels were significantly decreased between 4 and 8 h (*p* < 0.0001) and 4 and 48 h (*p* < 0.0001) post-treatment (Fig. 3A). However, 24 h after injection, they incurred in a significant and temporary increase compared to the 8-h (*p* < 0.0001) and 48-h (*p* < 0.0001) time points (Fig. 3A). At 48 h, 5-mC levels were significantly reduced compared to the 8-h time point (*p* < 0.0001), suggesting a dynamic and intricate response of the DNA methylation system to MPTP treatment. When comparing control and MPTP-injected mice significant differences were observed at 4 h (*p* < 0.0001) and 24 h (*p* < 0.0001), with higher 5-mC levels in the MPTP group. Conversely, at 48 h, 5-mC levels were significantly higher (*p* < 0.0001) in the control group compared to the MPTP group (Fig. 3B).

In the striatum of control mice, 5-mC levels followed a pattern similar to the MPTP group in the VM, showing a significant decrease between 4 and 8 h (*p* < 0.01) and 4 and 48 h (*p* < 0.001) post-treatment, as well as a temporary significant increase at 24 h compared to the 8-h (*p* < 0.001) and 48-h (*p* < 0.001) time points. At 24 h, 5-mC levels returned to values similar to those observed at 4 h post-saline injection (Fig. 3C). This pattern highlights a dynamic of DNA methylation under normal conditions in the striatum, with fluctuations over time. In the MPTP-treated group, a different temporal profile was observed (Fig. 3C). A sharp and significant decrease in 5-mC levels occurred between 4 and 8 h (*p* < 0.0001), followed by a return to the baseline levels at 24 and 48 h. At this later time points, 5-mC levels were significantly higher compared to the 8-h time point, (*p* < 0.0001), indicating a biphasic response to MPTP treatment, characterized by an initial decrease in DNA methylation followed by a sustained increase. Comparing control and MPTP-treated mice, significant differences were observed at 4 h (*p* < 0.01), 24 h (*p* < 0.001) and 48 h (*p* < 0.0001), with 5-mC levels consistently higher in the MPTP-treated group (Fig. 3D).

### Dynamics of 5-Hydroxymethylcytosine in Ventral Mesencephalon and Striatum Following MPTP Treatment

**Fig. 4:**
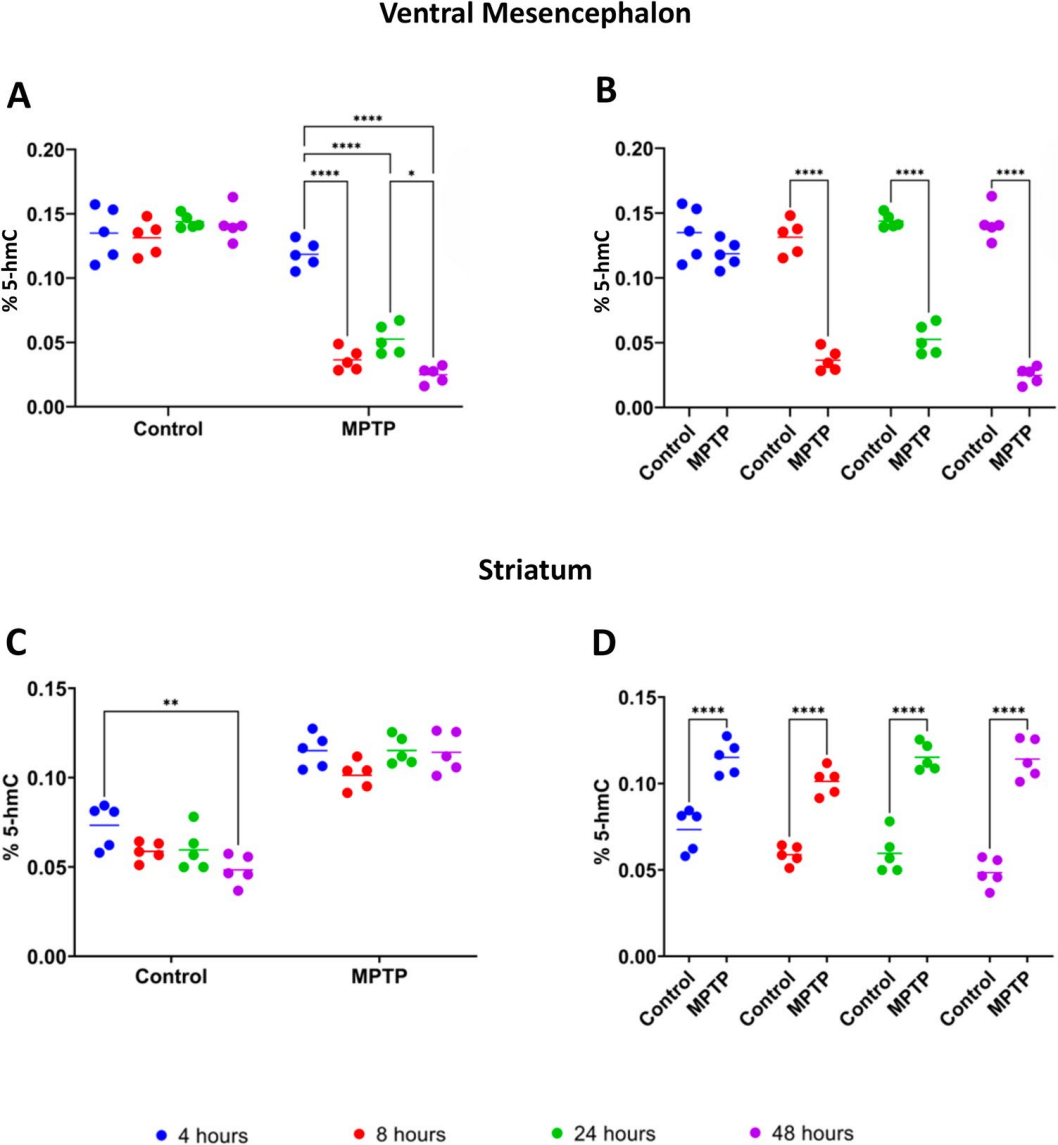
Levels of 5-Hydroxymethylcytosine (5-hmC) in the ventral mesencephalon (**A**,**B**) and striatum (**C**,**D**) of saline-(control) and MPTP-treated mice. **A** Evaluation of 5-hmC levels in the ventral mesencephalon within each experimental group across the different time points after. **B** Comparison of 5-hmC levels in the ventral mesencephalon between control and MPTP-treated groups at the different time points (**C**) Evaluation in 5-hmC levels in the striatum within each experimental group across the different time points after. **D** Comparison of the 5-hmC levels in the striatum between control and MPTP-treated groups at the different time points. Results were expressed as the mean ± SD of the percentage of 5-hmC relative to total DNA and evaluated for statistical significance by a two-way ANOVA followed by Tukey’s multiple comparison test. *p ≤ 0.05, **p < 0.01, ***p < 0.001, ****p < 0.0001

In the VM, the control group showed no statistically significant differences in 5-hmC levels over time (Fig. 4A). In contrast, the MPTP-treated group exhibited a significant decrease in 5-hmC levels between 4 and 8 h (*p* < 0.0001), 4 and 24 h (*p* < 0.0001) and 4 and 48 h (*p* < 0.0001) post-treatment (Fig. 4A). A significant decrease was also observed between 24 and 48 h (*p* < 0.05), suggesting a marked reduction in DNA hydroxymethylation following MPTP treatment. When comparing the control and MPTP-treated groups (Fig. 4B), significant decreases in 5-hmC levels were found in the MPTP-injected mice at 8 h, 24 h, and 48 h post-treatment (*p* < 0.0001).

In the striatum, the control group showed a significant decrease in 5-hmC levels only between 4 and 48 h (*p* < 0.01) (Fig. 4C), while no statistically significant changes were found in the MPTP-treated group over time. By comparing the two groups (Fig. 4D), significant increases in 5-hmC levels were found in the MPTP group compared to the control group at all evaluated time points (*p* < 0.0001). These findings indicate an early and persistent effect of MPTP treatment, leading to elevated 5-hmC levels in the striatum.

## Discussion

This study provides a comprehensive analysis of the effects of MPTP treatment on 8-OHdG, 5-mC, and 5-hmC levels in the VM and striatum of mice. The MPTP-induced mouse model is widely used to study PD pathogenesis, a neurodegenerative disorder characterized by multiple hallmarks, including neuroinflammation and oxidative stress (Barcia et al 2013; Costa et al 2021; Puspita et al. 2017; Lee et al. 2019; Dionisio et al. 2021; Chakrabarti and Bisaglia 2023). In this model, MPTP is metabolized into MPP +, which selectively targets dopaminergic neurons, leading to mitochondrial dysfunction, increased ROS production, and oxidative damage (Dionisio et al. 2021).

### Implication of 8-OHdG in the Response to MPTP Acute Treatment

Our results demonstrate that MPTP treatment induces early and pronounced oxidative stress in the VM, as evidenced by elevated 8-OHdG levels 4 h after injection. These levels progressively and significantly decrease at later time points, suggesting a persistent oxidative stress response that diminishes over time. However, in regions like the VM, where dopaminergic neurons are highly vulnerable to MPTP-induced damage and significantly depleted **(**Blesa et al. 2012; Annese et al. 2015), this reduction in 8-OHdG may also reflect neurodegeneration rather than neuron repair. Interestingly, Venkateshappa and colleagues (Venkateshappa et al. 2012) reported that during normal aging, the substantia nigra (SN) undergoes extensive oxidative damage, including loss of antioxidant and mitochondrial function and increased glial fibrillary acid protein expression. Moreover, changes in oxidative stress markers have been shown to precede significant neuronal loss and glial activation, suggesting that oxidative stress and inflammation are early events in the MPTP-induced neurodegenerative cascade (Gil-Martinez et al. 2020a). This heightened vulnerability to neurotoxic insults may contribute to the selective neurodegeneration observed in PD.

In the control group, a single saline injection resulted in a reduction of oxidative DNA damage at the 48-h time point, likely due to physiological repair mechanisms responding to injection-induced stress. This allowed for significant differences to emerge between the MPTP and control groups at later time points (24 and 48 h), emphasizing the sustained impact of MPTP on DNA oxidation.

The oxidative response to MPTP observed in the striatum, where dopaminergic fibers from the SN pars compacta project, also showed activation of 8-OHdG-driven mechanisms. However, unlike the VM, significant differences in the striatum were only detected between the 4-h time post-injection measurement and later time points (8, 24 and 28 h). This suggests that the oxidative response to MPTP in the striatum is less pronounced than in the VM, highlighting region-specific susceptibilities and adaptative mechanisms in response to oxidative stress. Similar regional differences have been reported in both PD patients (Mythri et al. 2011) and MPTP animal models (Sinha et al. 2020), reinforcing the idea that oxidative stress is modulated by the interplay between regional antioxidant capacity and vulnerability to damage. Interestingly, findings from the control group, also revealed a significant decrease in 8-OHdG levels over time in the striatum, which was more pronounced at 24 and 48 h compared to MPTP-treated mice. This suggests alternative hypothesis beyond the previously discussed mechanisms. One possibility is that MPTP alters key signaling pathways involved in the antioxidant response, such as the transcription factors Nrf2, which is typically activated in response to oxidative stress (He et al. 2020). A disruption of Nrf2 activation could impair the upregulation of protective genes, leading to decreased production of antioxidant enzymes. Another hypothesis considers the extensive cellular damage induced by MPTP, which may result in rapid depletion of antioxidant reserves ultimately leading to a diminished oxidative stress response compared to controls.

### Implication of 5-mC in the Response to MPTP Acute Treatment

As stated in the introduction, several studies have linked PD to epigenetic modifications (Kochmanski et al. 2022; Tsalenchuk et al. 2023). Among these, DNA methylation at the cytosine 5-carbon position (m-5 mC) and its oxidated form 5-hmC, have been identified as key regulatory markers in PD, as observed in the cortex, blood and saliva of PD patients (Chuang et al. 2017; Henderson et al. 2021; Kaut et al. 2022). However, it remains unclear whether these epigenetic changes also occur in major PD-affected brain regions such as the VM and striatum. To investigate this, we analyzed 5-mC and 5-hmC levels in these regions following the well-established MPTP-induced model of murine parkinsonism.

In the VM of control mice, we observed a significant increase in cytosine methylation at 8, 24 and 48 h compared to the 4-h time point. This suggests active regulation of DNA methylation following the injection protocol, confirming this area as highly susceptible to manipulation. This stability in methylation status may be essential for maintaining neuronal function and gene expression homeostasis in the VM. Conversely, in the MPTP-treated group, 5-mC levels significantly decreased between 4 and 8 h post-injection, followed by a transient increase at 24 h and a subsequent decrease at 48 h. This biphasic response suggests a complex interplay between oxidative stress and DNA methylation. The initial demethylation phase may be a consequence of oxidative stress, while the subsequent increase at 24 h could indicate an attempt at compensatory remethylation. The further reduction at 48 h might reflect the resolution of oxidative stress or epigenetic adaptation to neuronal loss. Overall, these dynamic changes in DNA methylation align with previous findings suggesting epigenetic regulation of genes associated with neuronal survival and plasticity under neurodegenerative conditions (Younesian et al. 2022).

In the striatum, 5-mC levels in control mice exhibited a biphasic response, with an initial decrease at 8 h, a transient increase at 24 h, and a subsequent decrease at 48 h. This suggests an active and dynamic regulation of DNA methylation in response to injection procedure. The different response compared to the VM may be related to regional differences in neuronal plasticity or adaptation to external stimuli.

In the MPTP-treated group, 5-mC levels initially decreased at 8 h, mirroring the the response observed in the VM, indicating an immediate disruption of DNA methylation. However, the restoration of 5-mC levels baseline levels at 24 and 48 h suggests a recovery or compensatory mechanisms unique to the striatum, possibly related to its distinct cellular composition and regional resilience. When comparing control and MPTP-treated groups at each time point, 5-mC levels were significantly higher in the MPTP group at all time point except 8 h. This suggests a persistent alteration in DNA methylation pathways in the striatum, potentially associated with the activation of stress-responses or neuroinflammatory genes. Several studies have emphasized the critical role of inflammatory processes mediated by microglia and astrocytes in the development and progression of PD (Hirsch and Hunot 2009; Pajares et al. 2020), particularly in aging brains, where cellular degeneration and glial dysfunction are more pronounced (Zhang et al. 2024). A neuroinflammatory role has been demonstrated in the MPTP mouse model of parkinsonism (Annese et al. 2015; Gil-Martinez et al. 2020a), where a prominent oligodendrogliosis has also been described (Annese et al. 2013). Therefore, the complexity and seemingly heterogeneous responses to MPTP treatment, as well as the experimental manipulation, suggest that a range of events involve not only neurons but also glial cells (Barcia et al 2013). Waves of pro-inflammatory responses (i.e. microgliosis, astrogliosis) and attempts at circuit restoration (i.e. oligodendrogliosis) overlap with neuronal responses to treatment, adding layer of complexity to the interpretation of results.

Our findings align with a growing body of evidence, highlighting the intricate interactions between neuroinflammation, oxidative stress, and dopaminergic neurodegeneration. A study by Gil-Martinez et al. (2020a) showed that MPTP-induced parkinsonism in aged mice (20 months old) exacerbated neuronal death and intensified glial responses in the nigrostriatal pathway. Furthermore, an unexpected increase in neuronal loss and glial activation was observed in aged MPTP-treated mice subjected to combined anti-inflammatory (HA-1077) and antioxidant (NAC) treatments, Highlighting the complexity of these processes (Gil-Martinez et al. 2018). This paradoxical effect, particularly linked to increased JNK phosphorylation in certain treatment groups, suggests that therapeutic interventions beneficial in younger models may have adverse effects in aged, more vulnerable brains (Gil-Martinez et al. 2018). These findings emphasize the importance of age-specific considerations when developing therapeutic strategies for PD.

### Implication of 5-hmC in the Response to MPTP Acute Treatment

When examining 5-hmC, no significant changes were observed in the control group of the VM. However, in the MPTP-treated group, a significant decrease in 5-hmC levels was evident at all evaluated time points. The significant differences between groups at 8, 24, and 48 h post-MPTP treatment suggest a dysfunction in the regulatory mechanisms governing DNA hydroxymethylation. This finding is consistent with previous studies demonstrating that oxidative stress impairs the function of ten-eleven translocation enzymes, which are responsible for converting 5-mC into 5-hmC (Al-Mahdawi et al. 2014). A decrease in DNA hydroxymethylation may contribute to neuronal vulnerability and neurodegenerative processes (Hwang et al. 2017; Xylaki et al. 2019).

In the striatum, a trend toward decreased in 5-hmC levels was observed between 4 and 8 h, as well as between 4 and 24 h, but this decrease became statistically significant only at 48 h post-injection, indicating minimal fluctuations in DNA hydroxymethylation over time. Interestingly, although no significant changes in 5-hmC levels were observed in the MPTP group across time points, when compared to controls, the MPTP-treated mice exhibited significantly higher 5-hmC levels at all time points. This suggests an early and persistent elevation of DNA hydroxymethylation in the striatum following MPTP exposure, potentially reflecting compensatory or adaptive mechanisms in response to oxidative stress aimed at mitigating neurotoxicity.

Overall, the differences in 5-hmC dynamics between VM and striatum highlight the complexity of epigenetic regulation in response to neurotoxic insults and underscore regional variations in vulnerability and adaptative responses.

## Conclusions

This study demonstrates that MPTP treatment induces dynamic and region-specific changes in 8-OHdG, 5-mC, and 5-hmC levels in the mouse VM and striatum. These changes can be categorized as early (4–8 h), transient (24 h) and late (48 h), reflecting complex responses to oxidative stress, neuroinflammation, and neurotoxicity. The rapid onset of these epigenetic and oxidative stress alterations, which precede neuronal and glial modifications observed in previous studies, highlights their potential as early biomarkers in the neurodegenerative cascade of PD. Moreover, the link between peripheral inflammation and central epigenetic and oxidative stress changes aligns with Braak's theory, which suggests a possible gut origin of PD (Braak et al. 2003). Notably, previous research by or group demonstrated that MPTP treatment of mice subjected to dextran sodium sulfate-induced experimental ulcerative colitis significantly exacerbated microglia and astrocyte activation, as well as dopaminergic neuron loss in the SN pars compacta (Gil-Martinez et al. 2019). This effect was further reflected in a distinct gene expression profile in mice subjected to combined treatments compared to those receiving single treatments (Costa et al. 2021; Gil-Martinez et al. 2020b).

Understanding this interplay between oxidative stress, epigenetic modifications, and neuroinflammation in PD could have the way for novel therapeutic strategies. Future research should explore interventions targeting both peripheral and central mechanisms, with the aim of mitigating neuronal damage and improving clinical outcomes in PD patients.

## Data Availability

The data supporting the findings of this study are available at: https://doi.org/10.6084/m9.figshare.28346948.v1.
